# Prevention of Tuberculosis

**Published:** 1905-02

**Authors:** 


### Prevention of Tuberculosis.

A meeting of physicians was held on January 6th in Chicago, to organize an Illinois Society for the Prevention of Tuberculosis. It is hoped to get a State appropriation of $250,000 for a State tuberculosis sanitarium. Outdoor camps are also to be established.

Mr. W. H. McClain, general manager of the St. Louis Provident Association has tendered to the Society for the Prevention of Tuberculosis the gift of a fund, by an anonymous donor, for the establishment of fresh air colonies for consumptives. A tract of land near Montesano Springs, Mo., on the Iron Mountain Railroad, forms part of the gift. The donor will erect thereon a hospital and many small pavilions.

At Santa Fe, N. M., the city council has decided to give six hundred and forty acres of land to the National Fraternities' Sanitarium for Consumptives.—*Med. Review.*

